# Digital PCR for direct quantification of viruses without DNA extraction

**DOI:** 10.1007/s00216-015-9109-0

**Published:** 2015-10-19

**Authors:** Jernej Pavšič, Jana Žel, Mojca Milavec

**Affiliations:** Department of Biotechnology and Systems Biology, National Institute of Biology, Večna pot 111, 1000 Ljubljana, Slovenia; Jožef Stefan International Postgraduate School, Jamova cesta 39, 1000 Ljubljana, Slovenia

**Keywords:** Digital PCR, Molecular diagnostics, Direct quantification, Viruses, Virus reference materials, Human cytomegalovirus

## Abstract

DNA extraction before amplification is considered an essential step for quantification of viral DNA using real-time PCR (qPCR). However, this can directly affect the final measurements due to variable DNA yields and removal of inhibitors, which leads to increased inter-laboratory variability of qPCR measurements and reduced agreement on viral loads. Digital PCR (dPCR) might be an advantageous methodology for the measurement of virus concentrations, as it does not depend on any calibration material and it has higher tolerance to inhibitors. DNA quantification without an extraction step (i.e. direct quantification) was performed here using dPCR and two different human cytomegalovirus whole-virus materials. Two dPCR platforms were used for this direct quantification of the viral DNA, and these were compared with quantification of the extracted viral DNA in terms of yield and variability. Direct quantification of both whole-virus materials present in simple matrices like cell lysate or Tris-HCl buffer provided repeatable measurements of virus concentrations that were probably in closer agreement with the actual viral load than when estimated through quantification of the extracted DNA. Direct dPCR quantification of other viruses, reference materials and clinically relevant matrices is now needed to show the full versatility of this very promising and cost-efficient development in virus quantification.

## Introduction

Real-time PCR (qPCR) is a very sensitive and specific method for quantification of DNA copies, and it is therefore used for a range of clinical applications in the detection and quantification of viruses [1]. DNA extraction before amplification is considered an essential step for the measurement of viral DNA, as it releases the DNA from the capsid and eliminates any PCR-inhibitory substances that are initially present in the matrix [1, 2]. However, different extraction kits provide different amounts of DNA and remove varying amounts of inhibitory substances, consequently reducing agreement on viral loads and hampering inter-laboratory comparability of data [3, 4].

Whole-virus reference materials for several viruses have been developed to normalise this quantification variability caused by DNA extraction and suboptimal qPCR assay efficiencies, and therefore to improve comparability of viral-load measurements between laboratories [5]. The viral loads of these reference materials are assigned in terms of the mean qPCR data collected across participants in collaborative studies, and they are expressed as DNA copies (cp)/millilitre or in international units (IU)/millilitre [6, 7]. Consequently, the actual virus concentrations of reference materials remain unknown due to the variable performances of DNA extraction kits and the drawbacks related to relative quantification using qPCR, such as suboptimal commutability of calibration material and variable assay efficiencies [2, 8].

Digital PCR (dPCR) is a new PCR-based technology that has already been successfully used for sensitive and reproducible quantification of several different viruses [9]. As it allows robust and precise quantification of nucleic acid copies without the need for any calibration curves and with higher resistance to inhibition [10], dPCR might be more appropriate for quantification of viral loads. However, dPCR is still susceptible to errors caused by DNA extraction, such as variable and suboptimal DNA yields [10].

The two most commonly used dPCR platforms are the QX100™ Droplet Digital™ PCR system (Bio-Rad Laboratories) and the Biomark^TM^ HD system (Fluidigm), and these were used here for direct quantification of two whole-virus materials of human cytomegalovirus (HCMV), without the use of DNA extraction. Measurement of the extracted DNA was also carried out to provide a comparison with direct quantification, to define the potential benefits of this novel dPCR approach.

## Materials and methods

### Whole-virus materials

The 1st WHO International Standard for Human Cytomegalovirus for Nucleic Acid Amplification Techniques (National Institute for Biological Standards and Control [NIBSC], code 09/162; ‘WHO material’) [6] was purchased from the manufacturer and stored at −20 °C. The WHO material was in 10 mM Tris-HCl buffer (pH 7.4) and 0.5 % human serum albumin. In the collaborative study of several laboratories using qPCR, the WHO material had been assigned a mean HCMV concentration of 5 × 10^6^ cp/mL, which was later transformed by the manufacturer into 5 × 10^6^ IU/mL. Samples no. 365029, no. 365030 and no. 365032 from the Society for Promoting Quality Assurance in Medical Laboratories (INSTAND) [7] were kindly provided by Heinz Zeichhardt and Hans-Peter Grunert, and were stored at −20 °C. The INSTAND materials were in differently diluted lysates from MRC-5 cell cultures infected with HCMV. The final nominal HCMV concentrations of the INSTAND materials, which were each derived from the consensus value from INSTAND Target Value Laboratories using qPCR, were 77,000 cp/mL for sample no. 365029 and 20,000 cp/mL for sample no. 365032, with sample no. 365030 serving as a negative control.

### Preparation of materials for DNA extraction and direct quantification

Prior to the DNA extraction and direct quantification, the WHO and INSTAND materials were initially resuspended in 1 mL double-distilled water to reach the nominal concentrations provided by the manufacturers (5 × 10^6^ IU/mL for the WHO material, 77,000 cp/mL for INSTAND sample no. 365029 and 20,000 cp/mL for INSTAND sample no. 365032). To compare the performances of the direct quantification with those of the extracted DNA across a wide dynamic range, the WHO material was volumetrically diluted 1×, 10× and 100× in double-distilled water, for nominal HCMV concentrations of 5 × 10^6^, 5 × 10^5^ and 5 × 10^4^ IU/mL. The INSTAND samples were not diluted. All of the aliquots of both of these materials were kept at 4 °C and used within 24 h, for both the DNA extraction and the direct quantification using dPCR.

### DNA extraction

Before DNA extraction of the WHO material, the samples diluted 1×, 10× and 100× in double-distilled water were volumetrically diluted a further 5× either in Acrometrix^®^ EDTA Plasma Dilution Matrix (Life Technologies, USA), to mimic a clinical matrix, or in phosphate-buffered saline (PBS), pH 7.4 (137 mM NaCl, 2.7 mM KCl, 8 mM Na_2_HPO_4_, 2 mM KH_2_PO_4_). Consequently, 5×, 50× and 500× WHO sample dilutions reached nominal HCMV concentrations of 1 × 10^6^, 1 × 10^5^ and 1 × 10^4^ IU/mL. Before DNA extraction, the INSTAND samples were not diluted in any matrix, and therefore, their nominal HCMV concentration remained the same as above for the direct quantification. The DNA extractions were performed using High Pure Viral Nucleic Acid kits (Roche), which we have previously defined as the most efficient manual extraction method in an assessment of three commercial DNA extraction methods from different manufacturers (our unpublished data). All of the DNA extractions were carried out in triplicate, according to the manufacturer protocol. The eluted DNA was stored at 4 °C and analysed using dPCR within 24 h.

### Preparation of WHO materials for direct virus quantification from human plasma

To directly quantify samples containing a clinically relevant matrix, the 1× WHO material was volumetrically diluted an additional 10× in 100, 33.3, 11.1 and 3.7 % Acrometrix^®^ EDTA Plasma Dilution Matrix (Life Technologies, USA), which thus resulted in 90, 30, 10 and 3.3 % (*v*/*v*) human plasma in these samples. The initial dilutions of human plasma were performed volumetrically in double-distilled water. Each of these samples was then directly transferred to one or both of the dPCR platforms to perform the direct virus quantification.

### Copy number measurement of extracted and non-extracted viral DNA using dPCR

For the copy number measurements of the extracted DNA and directly from the whole viruses, the QX100 and Biomark dPCR platforms were used. PCR assays that targeted the DNA polymerase gene of the human cytomegalovirus (*UL54*) of the HCMV genome were performed on both of these dPCR platforms using 600 nM primers and 200 nM FAM-BHQ1 hydrolysis probes [11]. The cycling conditions for both of these dPCR platforms were 2 min at 50 °C, 10 min at 95 °C, and 45 cycles of 15 s at 95 °C and 1 min at 60 °C. For the last step on the QX100 system, 10 min at 98 °C was added.

On the QX100 system, the 20-μL reaction volumes were composed of 10 μL 2× Master Mix (ddPCR™ supermix for probes; Bio-Rad), 1 μL *UL54* PCR assay, 1 μL double-distilled water and 8 μL sample. To reduce the variability caused by pipetting of these small volumes of reaction components, a large volume of the reaction mix was initially prepared in a single tube, which was then distributed into several tubes as 12 μL reaction mix. The samples for direct dPCR quantification were analysed in triplicate and were separately added to the 12-μL reaction mix, while the DNA extraction triplicates were each analysed in a single reaction, to maintain equal numbers of observations for both of these quantification approaches and to facilitate the statistical analysis. Data analysis was carried out using the QuantaSoft Analysis software, version 1.3.2.0 (Bio-Rad). Manually determined thresholds allowed simple distinction between positive and negative droplets and were placed at the amplitudes between 1500 and 2500, with the exception of directly quantified INSTAND samples, where the thresholds were determined at the amplitude of 6000, due to higher base fluorescence of the negative droplets. Only reactions with accepted droplet counts above 10,000 were considered. The droplet volume of 0.834 μL was taken into account when calculating the DNA copy numbers in the samples [12].

For the Biomark system with the WHO material, 2.1 μL of the extracted and non-extracted, and non-diluted and 10×-diluted, samples were analysed on qdPCR 37K™ Integrated Fluidic Circuits using 6-μL reactions comprising 1.5 μL 4× TaqMan^®^ Fast Virus 1-Step Master Mix (Applied Biosystems), 0.3 μL *UL54* PCR assay, 0.6 μL 20× GE Sample Loading Reagent (Fluidigm) and 1.5 μL double-distilled water. Due to the lower nominal virus concentrations for INSTAND sample no. 365029, 4 μL of the extracted and non-extracted samples was tested in 10-μL reactions on 12.765 Digital Array™ Integrated Fluidic Circuits. These reactions were carried out with 2.5 μL 4× TaqMan^®^ Fast Virus 1-Step Master Mix (Applied Biosystems), 0.5 μL *UL54* PCR assay, 0.5 μL 20× GE Sample Loading Reagent (Fluidigm) and 2.5 μL double-distilled water. For each array, a large volume of reaction mix was initially prepared in a single tube, followed by its distribution into several tubes containing either 3.9 μL reaction mix (6-μL reaction) or 6 μL reaction mix (10-μL reaction). The samples for direct quantification were analysed in duplicate and were separately added to the reaction mix. For the DNA extraction triplicates, only two randomly selected triplicates were analysed each in a single reaction, to provide equal numbers of observations as for the direct quantification, and therefore to allow better comparisons of variability between these two approaches. Data analysis was carried out using the Biomark™ HD Data Collection Software, v3.1.4 (Fluidigm), with manual determination of the fluorescence threshold, the accepted quantification cycle in real-time polymerase chain reaction (Cq) range (15–45 Cq) and the quality threshold (0.2).

### Statistical analysis

The coefficient of variability (CV) for the replicates was calculated using the formula:

1 $$ \mathrm{C}\mathrm{V}=\frac{\mathrm{standard}\;\mathrm{deviation}}{\mathrm{mean}\;\mathrm{concentration}} $$

Student’s *t* tests (two tailed, two-sample equal variance) were used in Microsoft Excel 2007 to determine the statistical significances of the differences between the different sets of measurements.

## Results

### Comparison of the direct quantification of DNA from whole viruses and quantification of extracted viral DNA

**Fig. 1:**
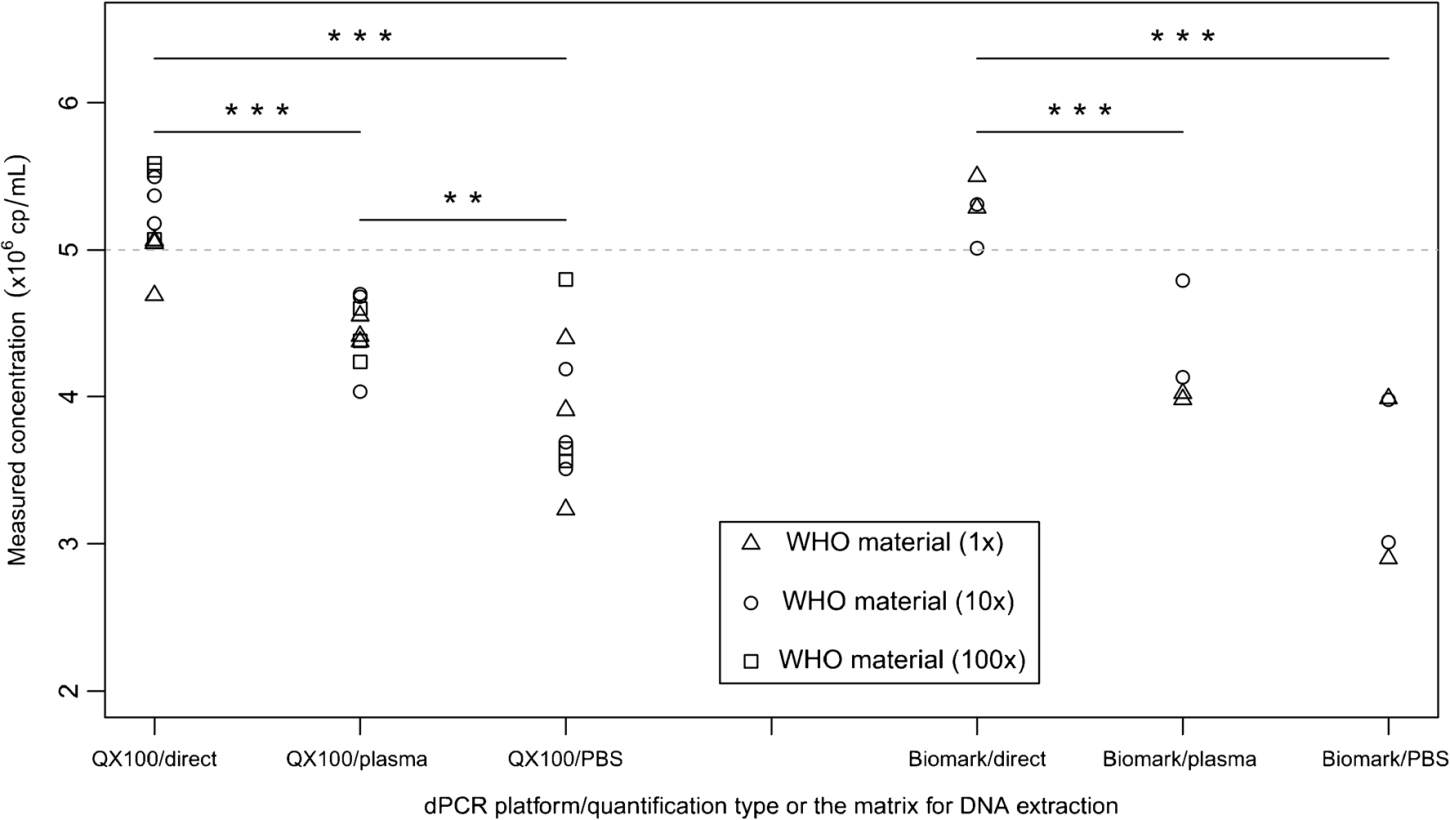
Comparison of direct quantification of DNA from whole-virus materials and quantification of extracted viral DNA using the WHO material on each of the dPCR platforms. The nominal predicted concentration is shown as a *grey dotted line*. Each *data point* represents a single dPCR measurement. For the DNA extraction, an additional 5× dilution in human plasma or PBS buffer was performed for each dilution of the WHO material. ***p < 0.001 and **p < 0.01 between the two groups for DNA copy numbers (Student’s *t* tests)

**Fig. 2:**
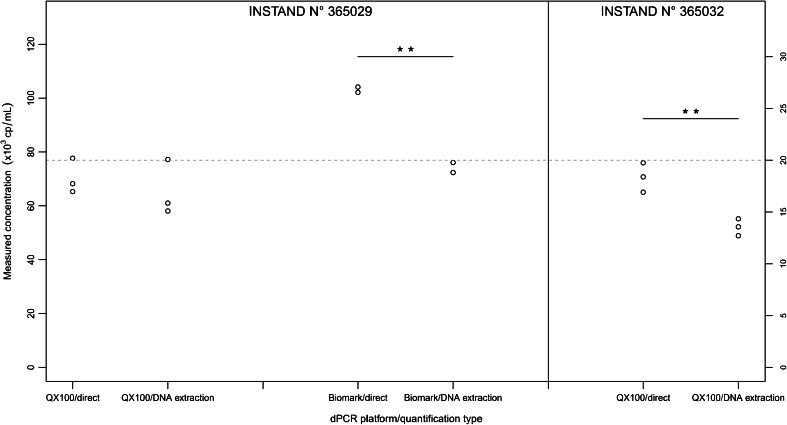
Comparison of copy number measurements between the direct quantification of DNA from whole viruses and the quantification of extracted viral DNA using the INSTAND material on each of the dPCR platforms. The nominal predicted concentration is shown as a *grey dotted line*. Each *data point* represents a single dPCR measurement. **p < 0.01 between the two groups for DNA copy numbers (Student’s *t* tests)

Both quantification approaches were assessed using both the QX100 and Biomark systems and using the WHO material and INSTAND samples. On the QX100 system that used direct quantification of the viral DNA, the DNA copy number measurements were 18 and 35 % higher in comparison to the quantification of the extracted DNA from human plasma and PBS buffer, respectively (Fig. 1). Even greater differences were seen using the Biomark system, with 26 and 53 % higher DNA concentrations observed, respectively. The same pattern was observed with INSTAND sample no. 365032 analysed using the QX100 system and INSTAND sample no. 365029 analysed using the Biomark system, where 39 and 35 % higher DNA concentrations were measured, respectively, using direct quantification (Fig. 2). In contrast, when INSTAND sample no. 365029 was analysed, using the QX100 system, no statistically significant differences were found between either of the quantification approaches.

### Comparison of DNA extractions from human plasma and PBS

Viral DNA was extracted from 1×, 10× and 100× dilutions of the WHO material that were then further diluted 5× in human plasma or PBS. On the QX100 system, extraction of the viral DNA from human plasma was more efficient compared with the extraction from PBS buffer, as the mean measured copy numbers were 15 % higher with the human plasma (*p* < 0.01) (Fig. 1). On the contrary, on the Biomark system, although 22 % higher mean measured copy numbers were seen for human plasma versus PBS, this difference did not reach statistical significance.

### Agreement between the dPCR platforms

No statistically significant differences were seen between the QX100 and Biomark systems for the mean measured copy numbers of the WHO material and INSTAND samples (Figs. 1 and 2), with the exception of direct quantification of INSTAND sample no. 365029, where the Biomark system showed 47 % higher mean measured copy numbers compared to the QX100 system (*p* < 0.01). However, the lack of statistically significant differences should be taken with caution here due to the small number of measurements performed with the WHO material on the Biomark system and with the INSTAND sample on both of the dPCR platforms.

### Assessment of variability

Direct quantification of the DNA derived from the whole virus particles was equally repeatable, or even more repeatable, than that of the extracted DNA, independent of the dPCR platform used or the material used (Tables 1 and 2). Additionally, the CVs using direct quantification did not exceed 10 %, while the quantification of the extracted DNA from the PBS buffer had CVs generally >10 %. On the Biomark system using extracted DNA from samples in PBS, higher variability was observed in comparison to that on the QX100 system.

**Table 1:** Variability of the direct quantification of DNA from whole viruses and quantification of extracted viral DNA using different materials on the QX100™ Droplet Digital™ PCR system. DNA extractions were performed using either WHO material spiked with human plasma and PBS or INSTAND samples with viruses in cell lysates

Material	Direct quantification			Quantification of extracted DNA						
	Nominal viral load^a^ (IU/mL or cp/mL)	*λ* ^b^	CV^c^ (%)	Nominal viral load^a^ (IU/mL or cp/mL)	Human plasma		PBS		Cell lysate	
					*λ* ^b^	CV^c^ (%)	*λ* ^b^	CV^c^ (%)	*λ* ^b^	CV^c^ (%)
WHO (1×)^d^	5 × 10^6^	1.6	4	1 × 10^6^	1.18	2	1	15	–	–
WHO (10×)^d^	5 × 10^5^	0.17	3	1 × 10^5^	0.12	8	0.1	9	–	–
WHO (100×)^d^	5 × 10^4^	0.017	5	1 × 10^4^	0.01	8	0.01	17	–	–
No. 365029^e^	7.7 × 10^4^	0.022	9	7.7 × 10^4^	–	–	–	–	0.08	20
No. 365032^e^	2 × 10^4^	0.006	8	2 × 10^4^	–	–	–	–	0.17	10

^a^Nominal viral concentration of the sample before quantification, based on the nominal concentration of each of the whole virus materials and their volumetric dilutions in double-distilled water, human plasma or PBS

^b^Mean DNA copy number per partition

^c^Coefficient of variability that was calculated on the basis of three measurements (*n* = 3) on the QX100 system

^d^First WHO International Standard for Human Cytomegalovirus for Nucleic Acid Amplification Techniques [6]

^e^INSTAND external quality assurance scheme sample [7]

**Table 2:** Variability of the direct quantification of DNA from whole viruses and quantification of extracted viral DNA using different materials on the Biomark^TM^ HD system. DNA extractions were performed using either WHO material spiked with human plasma or PBS, or INSTAND samples with viruses in cell lysates. WHO (100×) material and INSTAND sample no. 365032 were not analysed due to inadequate nominal DNA concentrations (<40 copies/effective reaction size)

Material	Direct quantification			Quantification of extracted DNA						
	Nominal viral load^a^ (IU/mL or cp/mL)	*λ* ^b^	CV^c^ (%)	Nominal viral load^a^ (IU/mL or cp/mL)	Human plasma		PBS		Cell lysate	
					*λ* ^b^	CV^c^ (%)	*λ* ^b^	CV^c^ (%)	*λ* ^b^	CV^c^ (%)
WHO (1×)^d^	5 × 10^6^	1.6	2	1 × 10^6^	1.18	3	1	25	–	–
WHO (10×)^d^	5 × 10^5^	0.15	5	1 × 10^5^	0.12	16	0.1	29	–	–
No. 365029^e^	7.7 × 10^4^	0.25	0	7.7 × 10^4^	–	–	–	–	0.08	5

^a^Nominal viral concentration of the sample before quantification, based on the nominal concentration of each of the whole virus materials and their volumetric dilutions in double-distilled water, human plasma or PBS

^b^Mean DNA copy number per partition

^c^Coefficient of variability that was calculated on the basis of two measurements (*n* = 2) on the Biomark system

^d^First WHO International Standard for Human Cytomegalovirus for Nucleic Acid Amplification Techniques [6]

^e^INSTAND external quality assurance scheme sample [7]

### Direct quantification from human plasma

**Fig. 3:**
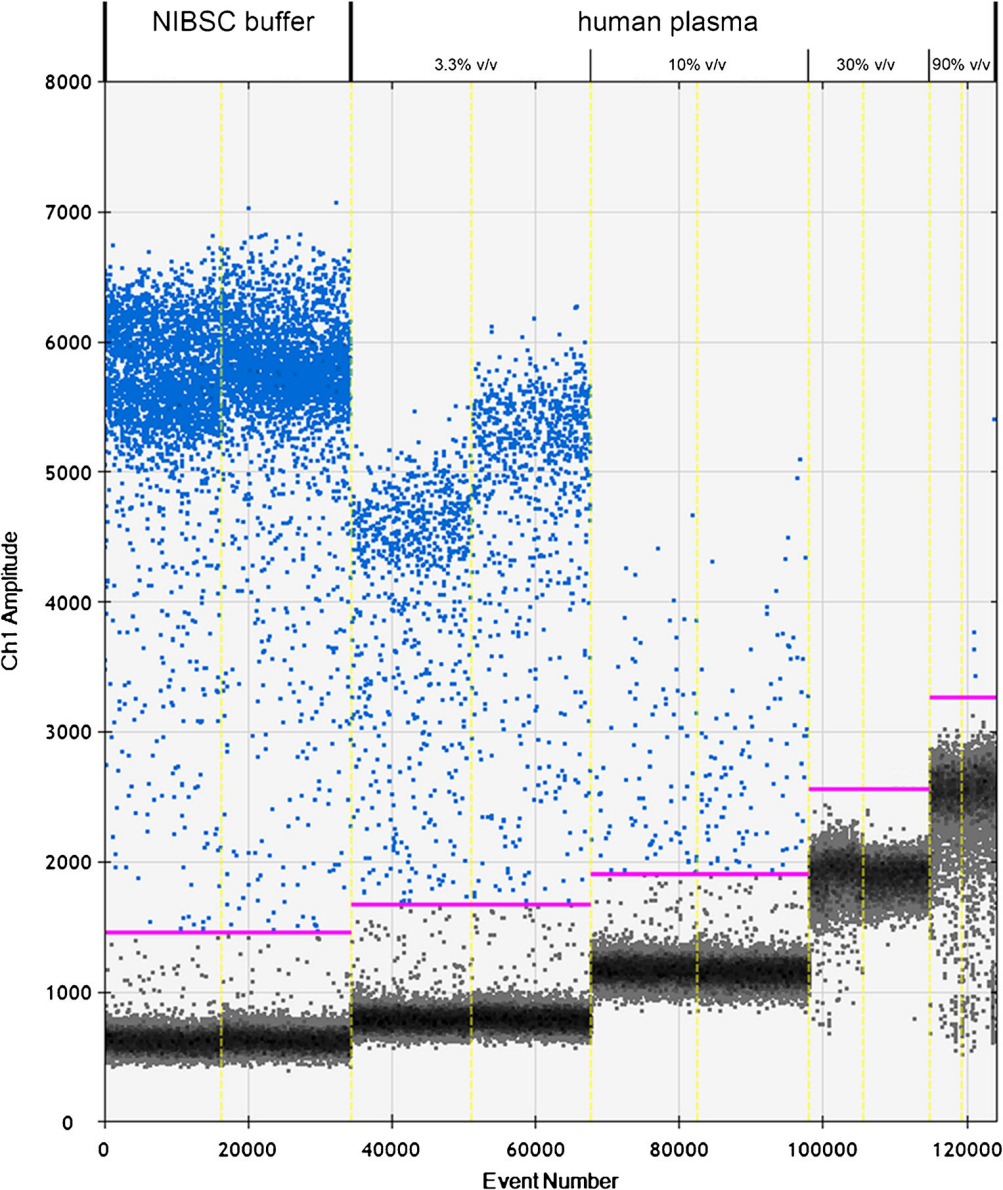
Determination of direct quantification of viral DNA from different matrices. The *left column* represents the direct quantification of the 10× WHO reference material in NIBSC buffer (10 mM Tris-HCl, pH 7.4, 0.5 % human serum albumin). The *columns on the right* represent the 1× WHO material spiked into different concentrations of human plasma (3.3, 10, 30, and 90 %; all *v*/*v*), which resulted in a 10× dilution of the initial material

Various effects on the direct quantification were seen for the samples with different human plasma concentrations (Fig. 3). For the samples with 3.3 and 10 % (*v*/*v*) human plasma, the measured copy numbers were only 31 and 4 % of the nominal concentrations, respectively. For duplicates with 3.3 % (*v*/*v*) human plasma, the CV value was 5 %, while for duplicates with 10 % (*v*/*v*) human plasma, the CV value was 11 %. On the other hand, no amplification was observed with samples containing ≥30 % (*v*/*v*) human plasma. Additionally, negative effects on the droplets were observed, as only around 4600 ± 200 droplets and 8300 ± 900 droplets were acceptable for analysis by the software for the samples with 30 and 90 % (*v*/*v*) human plasma, respectively. In contrast, there was no such influence with the lowest two concentrations of human plasma, for which around 16,000 droplets were analysed. The inclusion of human plasma influenced the intensity of the fluorescence, as in the positive droplets it was inversely proportional to the concentration of the matrix, while the opposite effect was seen for negative droplets.

## Discussion

To the best of our knowledge, this is the first report of direct quantification of DNA derived from whole virus particles performed on two different dPCR platforms. This study indicates that the heat treatment at 95 °C during dPCR was enough to degrade the HCMV viral coat proteins and to release the viral DNA into the reaction mixture. Although some viruses have already been directly quantified using qPCR without DNA extraction, such as HCMV, hepatitis B virus and human adenovirus [13–15], drawbacks related to the qPCR formats probably hampered the precise and reliable measurements of the actual virus concentrations using this direct quantification approach, such as susceptibility to inhibitory substances and dependence on the calibration material [2]. The commutability of such calibration material with clinical samples would probably represent an additional problem, as the calibration materials do not always behave in the same way as whole-virus-derived DNA from different matrices, which can lead to overestimation or underestimation of the DNA in the samples tested [8].

In the present study with the direct quantification approach using whole-virus materials in simple matrices, higher DNA copy numbers were measured compared to those from the extracted DNA, regardless of the dPCR platform used. In contrast to qPCR, dPCR is more resistant to inhibitory effects of different matrices and is not dependent on calibration material and its commutability [10], and thus only minor overestimation and underestimation of the actual DNA copy numbers are expected. Consequently, the differences in the copy number measurements between these approaches are most likely to be due to DNA loss during the DNA extraction. Therefore, it is reasonable to assume that the direct quantification measurements will be in closer agreement with the actual virus concentrations than the quantification of the extracted DNA.

Although in most cases good agreement was observed between the direct virus quantification and the nominal virus values for both of the whole-virus materials, the potential for bias of dPCR measurements still needs to be taken into consideration, as the nominal concentrations of each of these materials might not be close to the actual viral load, as they were assigned in collaborative studies using different qPCR assays and extraction kits [6, 7]. Therefore, it is difficult to determine the levels of agreement between the data based on the measured copy numbers of amplified DNA derived from whole viruses and the actual viral load in the sample or the reference material. As overestimation can occur only when two single-stranded DNA molecules that derive from the same double-stranded DNA are found in two different partitions [10], this is less likely to occur with direct quantification, as the DNA molecules are most likely packed inside the viral capsid at the time of partitioning. On the other hand, special care needs to be taken to define the possible sources of underestimation. In the case of molecular dropout, the DNA template is present but it is not amplified, due to factors that damage the DNA target region or make it less accessible to primers and DNA polymerase [10, 16]. Due to the presence of viral coat proteins and undiluted matrix, molecular dropout is probably more likely to occur for direct virus quantification than in pure DNA extracts. This phenomenon can be avoided by using two or more different assays that target various genomic regions on the DNA template, which will increase the probability that at least one region will be accessible and amplifiable [16]. Another possible source of underestimation is a non-homogeneous distribution of viruses caused by their linking prior to and during partitioning, as some viruses tend to aggregate [15]. As aggregation is dependent on the solution pH and the concentrations of specific cations and anions [15], various combinations of master mixes and matrices might result in different levels of aggregation. Therefore, the Biomark system can be used for the selection of the master mix composition that shows the highest disturbance of virus aggregation in specific matrices. Additionally, aggregation can potentially be regulated by the addition of cations and anions, or by adjusting the matrix pH. However, no virus-virus complexes have been observed for HCMV, to the best of our knowledge. Another possible factor for underestimation is the presence of dPCR inhibitors, such as human plasma and ethylenediaminetetraacetic acid (EDTA), which is discussed further below.

Although DNA extractions from human plasma on the QX100 system have been found to cause significantly higher DNA extraction yields than DNA extractions from PBS, on the Biomark system no statistical significance was found between these two matrices. However, this lack of statistical significance on the Biomark system might be due to the low number of measurements, as only two dilutions of WHO material were each tested in duplicate (*n* = 4), while on the QX100 system, three dilutions of the WHO material were each tested in triplicate (*n* = 9). Furthermore, as the High Pure Viral Nucleic Acid kits are designed for DNA extraction of viruses from human plasma, serum and whole blood, it appears likely that the DNA extractions from human plasma resulted in lower variability and higher DNA extraction yields than those from PBS. It could be speculated that in contrast to PBS, human plasma contains molecules, which in combination with extraction-kit buffers function as DNA carriers or prevent DNA molecules from fragmentation. This finding might indicate that the matrix had an important impact on the final measurements of the extracted DNA copy numbers. The different matrices will probably mainly alter the DNA extraction yields, as unsuccessfully eliminated PCR inhibitors would have only minor effects on the final DNA copy number estimations when dPCR is used [17, 18]. The differences between direct quantification and quantification of extracted DNA in terms of estimated DNA copy numbers might therefore allow assessment of DNA extraction by the evaluation of the matrix influence on the extraction yield. Thus, it appears reasonable to assume that the INSTAND material that comprised lysates of HCMV-infected MRC-5 cell cultures resulted in lower DNA extraction yields than the WHO material in human plasma, as greater differences were seen for the INSTAND material between the two quantification approaches.

Interestingly, equal performances for both of the quantification approaches were observed when INSTAND sample no. 365029 was measured on the QX100 system, in terms of the measured viral DNA copy numbers. As the statistically significant difference between both dPCR platforms was observed only with direct quantification of this particular sample, different matrix/master mix effects can be assumed, leading either to underestimation of the QX100 system or overestimation of the Biomark system. This hypothesis is additionally strengthened as there was no statistically significant difference between either of the dPCR platforms when the extracted DNA from INSTAND sample no. 365029 was measured, with no original matrix present. Furthermore, a 4× more diluted matrix might explain why no such matrix/master mix effects were seen with direct quantification of INSTAND sample no. 365032 [7]. Overestimation by the Biomark system or underestimation by the QX100 system due to partition volume variations is also not very likely, as no statistically significant differences were seen between these two platforms when measuring the WHO material. Therefore, as for all of the other samples tested on both of the dPCR platforms, statistically significant increases were observed when direct quantification was used instead of when extracted DNA was used; it is more reasonable to assume underestimation by the QX100 system than overestimation by the Biomark system.

In the majority of cases, the repeatability of the direct quantification was not only equal to or better than that for the DNA extracts, but also sometimes even lower than the theoretically predicted variability for both of these dPCR platforms using Poisson distributions [19]. This strongly indicates that the presence of whole viruses and different matrices does not influence the repeatability, while no additional variability is introduced as the DNA extraction step is avoided. Additionally, in the case of the extracted DNA from the WHO material in PBS, the higher variability that was noted with the Biomark system in comparison to that with the QX100 system might be due to the smaller numbers of measurements and/or smaller volumes of the sample input.

Although the chip-based Biomark system and the droplet-based QX100 system have both been shown to be more resilient to a range of inhibitors when compared to qPCR [17, 18], and might therefore be more appropriate for direct quantification from clinically relevant matrices, different inhibitory effects were observed with direct quantification from human plasma. Using the QX100 system, at the higher concentrations of human plasma, the formation of droplets was probably altered, as smaller numbers of droplets were accepted for analysis by the reader. Moreover, at all concentrations of human plasma, higher base fluorescence in the accepted droplets was observed, which might indicate a larger mean volume of the droplets in comparison to those without human plasma. This phenomenon probably correlates with the concentration of human plasma, as the highest base fluorescence was noted in droplets that contained the most concentrated human plasma. Inhibition not only reduced the fluorescence intensity in positive droplets but also hampered the final measurements of the DNA copy numbers. This is in partial agreement with a previous research conducted with the extracted HCMV DNA on the Biomark system, where in the presence of 25 % (*v*/*v*) human plasma, there was a very repeatable, approximately 10× decrease in the DNA copy number measurements [18]. However, more than a 20× decrease was seen in the present study when 10 % (*v*/*v*) human plasma was used, while there was no amplification for samples with 30 % (*v*/*v*) and 90 % (*v*/*v*) human plasma. The higher degree of inhibition in the present study might be the consequence of the presence of the anticoagulant, as 50 mM Na_2_EDTA, to which dPCR is very susceptible [17, 18]. Although EDTA can cause changes of up to 100× in the measurement of DNA copy numbers on dPCR [18], no such effect was seen for the two pairs of duplicates in the present study, although higher variability might have been detected if more replicates had been analysed. Direct quantification on either dPCR platform did not appear to be suitable for the quantification of HCMV from human plasma due to partial or complete PCR inhibition that resulted in underestimation of DNA copy numbers. However, successful qPCR-based quantification of HCMV from urine samples and hepatitis B from human serum have indicated the potential suitability of direct quantification of HCMV and other viruses from these two clinical matrices [13, 14].

## Conclusions

Direct quantification by dPCR is shown here to provide repeatable measurements of viral DNA copy numbers that appear to be in closer agreement with the actual viral load than with either dPCR-based or qPCR-based quantification of extracted DNA. This indicates that direct quantification of whole HCMV DNA using dPCR is a valuable approach for the characterisation of viral reference materials and materials for external quality assurance schemes that are in simple matrices, while unpromising results were obtained using the complex matrix of human plasma. Additionally, this direct quantification might also be appropriate for efficiency assessments of different extraction methods from simple matrices. Further studies using different assays and master mixes can now be performed to characterise and reduce the possible sources of underestimation. Exploration of direct quantification by dPCR for other viruses, reference materials and clinically relevant matrices is also necessary, to determine the full versatility of this very promising development in virus quantification.

## Abbreviations

Cq: Quantification cycle in real-time polymerase chain reaction
CV: Coefficient of variability
dPCR: Digital polymerase chain reaction
EDTA: Ethylenediaminetetraacetic acid
HCMV: Human cytomegalovirus
INSTAND: Society for Promoting Quality Assurance in Medical Laboratories
IU: International units
NIBSC: National Institute for Biological Standards and Control
PBS: Phosphate-buffered saline
qPCR: Real-time polymerase chain reaction
*UL54*: DNA polymerase gene of the human cytomegalovirus
WHO: World Health Organization
